# Health literacy of people living with HIV in a rural area in Indonesia: A cross‐sectional study

**DOI:** 10.1111/hsc.13075

**Published:** 2020-07-10

**Authors:** Elfride I. Sianturi, Dyah A. Perwitasari, Sitti N. Soltief, Md. Atiqul Islam, Bas Geboers, Katja Taxis

**Affiliations:** ^1^ PharmacoTherapy, ‐Epidemiology & ‐Economics (PTEE) Department of Pharmacy University of Groningen Groningen The Netherlands; ^2^ Faculty of Mathematics and Natural Sciences University of Cenderawasih Papua Jayapura Papua Indonesia; ^3^ Faculty of Pharmacy University of Ahmad Dahlan Yogyakarta Indonesia; ^4^ Rumah Sakit Umum Jayapura Papua Indonesia; ^5^ Department of Statistics Shahjalal University of Science and Technology Sylhet Bangladesh; ^6^ Department of Health Sciences University Medical Center Groningen University of Groningen Groningen The Netherlands

**Keywords:** beliefs, health literacy, HIV, Indonesia, Papua, stigma

## Abstract

Indonesia, the fourth most populated country in the world, has experienced a fivefold increase in Human Immunodeficiency Virus (HIV)‐infected individuals since 2001. Little is known about health literacy in people living with HIV (PLHIV) in Indonesia. This study aimed to determine the level of health literacy among PLHIV in Indonesia and assess associations between sociodemographic variables, beliefs about medicines, stigma and health literacy. We conducted a cross‐sectional study using questionnaires in PLHIV in Papua, Indonesia. The short version of the Test of Functional Health Literacy in Adults (S‐TOFHLA), Beliefs about Medicines Questionnaire (BMQ) and HIV stigma scale as well as questions on demographic information were completed by the participants from two hospitals in Papua, Indonesia. In a multivariate logistic regression analysis, we assessed the association between sociodemographic variables, stigma, beliefs about medicine and low health literacy. Overall, 331 participants were included, 62.0% female, 67.0% Papuans. A total of 38.5% of participants had low health literacy. PLHIV with multi‐dose regimen were less likely to have low health literacy than those taking a fixed‐dose combination (OR = 0.51; 95%CI = 0.32–0.82). PLHIV who had social support in medicine‐taking were more likely to have low health literacy (OR = 1.78; 95%CI = 1.07–2.97). More awareness about medication overuse (OR = 1.17; 95%CI = 1.06–1.29) and medication harm (OR = 1.10; 95%CI = 1.01–1.20) were also associated with having low health literacy. Overall, interventions targeting health literacy may be a promising strategy to improve self‐management.

**What is known about this topic?**

- The level of non‐adherence among people living with HIV in Indonesia was high particularly in rural area such as Papua Province.
- Low health literacy in people living with HIV (PLHIV) is associated with lower medication adherence and clinical outcomes including higher viral loads and more hospitalisations.
- It is important for health professionals to understand their patient's health literacy when providing care for PLHIV.

**What this paper adds?**

- More than a third of PLHIV in Papua have a low level of health literacy.
- Social support and giving a fixed‐dose combination seems to be important for PLHIV with low health literacy.
- Health providers should tailor their care to address concerns about medication overuse and harm, especially in PLHIV with low health literacy.

## 1 INTRODUCTION

Health literacy is increasingly recognised as an important determinant for health and illness behaviour among people living with HIV (PLHIV) (Gazmarariana et al., 2003; Palumbo, 2015). Although definitions vary, health literacy is commonly seen as the degree of competence in accessing, understanding, appraising and applying health‐related information (Sørensen et al., 2012). Studies in PLHIV have shown that low health literacy is associated with less knowledge about HIV and its treatment, lower medication adherence, and clinical outcomes including higher viral loads (Kalichman et al., 2013; Waldrop‐Valverde et al., 2009; Zukoski, Thorburn, & Stroud, 2011), and more hospitalisations (Palumbo, 2015). It is important for health professionals to understand their patient's health literacy when providing care (Raynor, 2012), particularly in tailored counselling for PLHIV where factors like stigma may also play a role (Pane et al., 2018). The level of health literacy may be an important factor to be taken into account when developing interventions to improve healthcare (Palumbo, 2015). Examples include better labels to reduce misunderstanding regarding medication‐taking behaviour (Davis et al., 2006). The majority of research on health literacy in PLHIV has been conducted in the United States, Europe and some regions in Africa (Wawrzyniak & Ownby, 2013). So far, little is known about health literacy among PLHIV in developing countries in Asia like Indonesia (Rajah, Hassali, & Murugiah, 2019)^.^

Indonesia, one of middle‐income countries in Asia, has experienced a fivefold increase in the number of PLHIV (Pendse, Gupta, Yu, & Sarkar, 2016), and HIV death rates (Murray, Ortblad, Guinovart, & Al, 2014) in the last 20 years. Currently, the prevalence is estimated to range between 0.1% and 2% across the country with Papua, one of the provinces in Indonesia, having the highest level of HIV prevalence in Asia (Koirala, Deuba, Nampaisan, Marrone, & Ekstro, 2017; Pendse et al., 2016). In recent years, unprotected sexual intercourse is thought to be the primary mode of transmission of HIV (Wijayanti et al., 2016). Even though antiretroviral therapy (ART) has transformed HIV from a fatal disease to a chronic disease, so far less than 20% of PLHIV in Indonesia were on ART (Koirala et al., 2017). The issue of low adherence (Sianturi, Perwitasari, Islam, & Taxis, 2019) and the limited range of ART regimens being available (Kementerian Kesehatan Republik Indonesia ‐ Direktorat Jenderal Pengendalian Penyakit dan Penyehatan Lingkungan, 2011) remain as problems among PLHIV in Indonesia.

The latest report shows that over 93% of Indonesians were literate (Indonesia S, 2013), and internet users (Asosiasi Penyelenggara Jasa Internet Indonesia, 2017). Studies have shown that PLHIV use the internet to obtain information to make health decisions (Perazzo, Haas, Webel, & Voss, 2017) and as support to deal with depressive symptoms of being infected with HIV (van Luenen, Garnefski, Spinhoven, & Kraaij, 2018). Previous studies showed that health literacy was associated with sociodemographic variables such as gender, age, level of education, and race (Waite, Paasche‐Orlow, Rintamaki, Davis, & Wolf, 2008). Resources, such as social support and capabilities, such as cognitive functioning also influence a person's health literacy skills (McCormack, Squiers, Boudewyns, Berkman, & Peinado, 2012). Beliefs about medicines have been significantly associated with health literacy in different diseases, locations and settings (Duggan et al., 2014; Kale et al., 2015). Misconceptions about medicines tend to occur among people with low health literacy. Stigma remains a problem related to HIV and is negatively associated with seeking access to treatment and care. The fear of stigma causes PLHIV to skip their ART when they feel that taking ART will disclose their status to others (Ware et al., 2009). In that way, stigma also influences PLHIV in making health decisions. The associations between health literacy and sociodemographic variables, stigma and beliefs about medicines need to be investigated in our setting since those associations depend on the setting and diseases (Gazmarariana et al., 2003). Therefore, this study aims to determine the level of health literacy among PLHIV in Indonesia and assess associations between sociodemographic variables, beliefs about medicines, stigma and health literacy.

## 2 METHODS

### 2.1 Study design, setting and participants

This study was approved by the Ethics Commission, Faculty of Medicine, Public Health, and Nursing Universitas Gadjah Mada, Indonesia (number KE/FK/1108/EC/2016). This cross‐sectional study utilised questionnaires on health literacy, beliefs about medicines, stigma and demographic characteristics in PLHIV. Data collection was conducted between September and November 2016. The participants were recruited among outpatients of two hospitals, one public hospital and one private hospital, in Jayapura, located in the province of Papua, Indonesia. We used convenience sampling and have chosen those two hospitals because they have more than two decades of experience in providing care for PLHIV, including diagnosis, treatment, laboratory monitoring and counselling. All PLHIV visiting in those two hospitals were considered as the total sample in our study. Treatment with ART in the hospitals is based on the national guideline (Kementerian Kesehatan Republik Indonesia ‐ Direktorat Jenderal Pengendalian Penyakit dan Penyehatan Lingkungan, 2011). In general, PLHIV received a multi‐dose regimen of ART. However, the guideline recommends a fixed‐dose combination of ART for patients with co‐morbidities such as tuberculosis (Kementerian Kesehatan RI, 2014).

Patients returned to the hospital monthly for their ART. An exception was made for PLHIV who lived in remote areas and had to fly to collect their medication, they received a supply for 3 months. The Indonesian government provides ART free‐of‐charge to PLHIV. Participants were included if they were at least 18 years old, were on ART for more than 6 months, collected their medication monthly at the outpatient clinic, were able to read and had basic numeracy skills, and signed informed consent. Participants for whom over 50% of data were missing were excluded from the analysis.

### 2.2 Instruments

#### 2.2.1 Health literacy

Health literacy was assessed using The Short Test of Functional Health Literacy in Adults (S‐TOFHLA). This has sufficient psychometric properties (Jordan, Osborne, & Buchbinder, 2011). The questionnaire measures functional health literacy and consists of two parts, a reading recognition test and a numeracy skills test. The first part consists of 36 multiple choice items which measure reading comprehension with questions about diseases, insurance and patient's knowledge about their rights to access healthcare (Parker, Baker, Williams, & Nurss, 1995). The second part consists of four questions which assess numeracy skills. We modified these four questions from the original S‐TOFHLA to fit the situation of HIV patients. A total score is calculated by adding up all individual items of the reading comprehension part. The total score ranges between 0 and 36, with a higher score indicating better health literacy. A total score of <23 was classified as low health literacy, a score of ≥23 was classified as high health literacy, based on previous studies (Kale et al., 2015) and this was based on sensitivity analysis. This was used as the main outcome measure in the statistical analysis (see below). We analysed the results from the numeracy assessment only descriptively.

#### 2.2.2 Beliefs about Medicines Questionnaire (BMQ)

The BMQ was developed to assess the concept of patient's beliefs about medication use in general and their beliefs about their own medication using a total of 18 questions (Horne & Weinman, 1999). All questions were scored on 5‐point Likert‐type scale (*1* = *strongly disagree, and 5* = *strongly agree*). A score is calculated for each of the four subscales by summing up the scores of the individual items. The BMQ‐General consists of an overuse and a harm subscale, with four questions each. The BMQ‐Specific has two subscales on necessity and concerns with five questions each. Higher total scores on the necessity subscale indicate that patients are able to see the advantages of taking their medication. Higher total scores on the concern, overuse and harm subscales indicate that patients have a negative perception of these aspects of medication use.

#### 2.2.3 HIV stigma

The HIV Stigma‐Sowell scale consists of three types of stigma, namely distancing, blaming and discrimination (Sowell et al., 1997). Items are scored on a 4‐point Likert‐type scale (1 = *not at all and 4 = often*). The distancing and blaming subscales were assessed with four questions each and discrimination was assessed with five questions. The total score ranges from 13 to 52 where a higher total score indicates a higher level of stigma.

### 2.3 Translation

All original instruments, BMQ, HIV stigma and S‐TOFHLA, were developed and written in English. Forward translation into target language, Bahasa Indonesia, was done by two Indonesian certified translators. The backward translation into English was carried out by an English native speaker to check the accuracy. All translators had no information about the original versions. Both versions of forward translation were assessed by one of the co‐authors who was very experienced in translating questionnaires. Finally, a reconciled Bahasa Indonesia version was agreed upon. The backward translation was modified several times because the target language does not recognise verb tenses (Epstein, Santo, & Guillemin, 2015). Therefore, the final version included words related to time. In a pilot project involving 47 PLHIV who did not participate in the main study, the questionnaires were tested. Besides assessing the intelligibility of the questionnaires, the internal consistency of the instruments was tested by calculating the Cronbach alpha. The Cronbach's alpha was .93 for S‐TOFHLA scale. Also the HIV‐stigma scale and the BMQ showed acceptable Cronbach alpha values (Sianturi et al., 2019). The pilot data indicated that participants understood the questionnaires.

### 2.4 Data collection

Patients were recruited by one pharmacist and nurses working at the two hospitals. The recruiters informed patients about the study while they were collecting their ART in the hospital. They emphasised that all information would be kept confidential and that the decision to participate or to refuse did not affect their treatment in any way. After the participants signed the informed consent, an appointment to complete the questionnaire was arranged for the following month as part of their next visit to the hospital. At the appointed time, participants were handed out the three paper‐based questionnaires and a questionnaire asking for the following sociodemographic information: age, gender, employment (unpaid, paid), marital status (single/widow, married), ethnicity (Papuan, non‐Papuan), ART regimen (fixed‐dose combination (FDC), multi‐dose regimen), and education (low education: primary school; intermediate education: 12 years formal education; advanced education: university level education). We also asked PLHIV whether they had someone who helped them with their medication, e.g. reminded them to take their medication. We categorised this as not having/having medicine‐taking support. Participants completed the questionnaires in the waiting area of the hospital. The recruiters were available for questions. Information about age, and type of ART including the type of regimen (fixed‐dose combination or multi‐dose regimen) was collected from the medical records. We attempted to collect data on patient's co‐morbidities from the medical records as well, but this information was not systematically recorded.

### 2.5 Statistical modelling

The data were analysed in Statistical Package for the Social Sciences (SPSS) version 23.0 for Windows. We used chi‐square tests for categorical variables and independent *t*‐test or Mann–Whitney *U* tests (when data were not normally distributed) for continuous variables to compare variables such as sociodemographic characteristics, BMQ and HIV stigma among PLHIV with low and high health literacy. The covariates with *p*‐value <.20 (Sharma & Jain, 2014) in univariate analyses were directly included into the multivariate logistic regression analysis. In the multivariate logistic regression analysis, we used a backward elimination procedure to select the final model with all independent variables being significant at *p*‐value <.05. Low health literacy as defined above was the outcome measure. Finally, odds ratios of independent variables and 95% confidence intervals were presented.

## 3 FINDINGS

We identified 1,305 people, with HIV positive status treated in the two study hospitals since 2006. We excluded patients who were lost during follow‐up and patients who lived far away from the hospitals. There were 360 PLHIV who were eligible to be the participants in this study. We excluded 29 participants who had more than 50% missing data. Finally, 331 participants were included in the analysis. The majority of PLHIV (62%) were female and married (60%) (Table 1). The mean age of the participants was 33.3 years (*SD* = 9.4) and more than half of the participants (66%) had someone supporting them in medicine‐taking. More participants, (67%) were of Papuan than non‐Papuan ethnicity. Slightly more than half of the participants (55%) used a multi‐dose combination, and reported having paid employment (58.0%). Overall, 38.5% of PLHIV had low health literacy. PLHIV with low health literacy also had low numeracy skills (Table 2).

**TABLE 1:** Sociodemographic characteristics of PLHIV with low and high health literacy

Independent variables		Total (*N*, %)	Low health literacy, *N* = 132 (38.5%)	High health literacy, *N* = 199 (61.5%)	*p*‐value
Age (mean ± *SD*)		33.3 ± 9.4	32.8 ± 9.7	33.6 ± 9.2	
	18–27 years	96 (29%)	42 (31.8%)	54 (27.1%)	.617
	28–37 years	135 (41%)	53 (40.1%)	82 (41.2%)	
	≥ 38 years	100 (30%)	37 (28.1%)	63 (31.7%)	
Gender	Male	127 (38%)	59 (44.6%)	68 (34.1%)	.054
	Female	204 (62%)	73 (55.4%)	131 (65.9%)	
Employment	Unpaid	139 (42%)	55 (41.6%)	84 (42.2%)	.922
	Paid	192 (58%)	77 (58.4%)	115 (57.8%)	
Marital status	Single/widow	131 (40%)	49 (37.1%)	82 (42.2%)	.457
	Married	200 (60%)	83 (62.9%)	117 (58.8%)	
Ethnicity	Papuan	222 (67%)	94 (71.1%)	128 (64.3%)	.192
	Non‐Papuan	109 (33%)	38 (28.9%)	71 (35.7%)	
Type of ART	Fixed‐Dose Combination (FDC)	148 (45%)	69 (52.2%)	75 (37.6%)	.009
	Multi‐dose regimen	183 (55%)	63 (47.8%)	124 (62.4%)	
Medicine‐taking support	No	113 (34%)	37 (28.0%)	76 (38.1%)	.056
	Yes	218 (66%)	95 (72.0%)	123 (61.9%)	
Education	Low	105 (32%)	46 (34.8%)	59 (29.6%)	.396
	Intermediate	171 (52%)	68 (51.5%)	103 (51.7%)	
	Advanced	55 (16%)	18 (13.7%)	37 (18.7%)	

Abbreviations: ART, antiretroviral therapy; FDC, fixed‐dose combination; *N*, number of participant; *SD*, Standard Deviation.

**TABLE 2:** Percentage of correct responses among 331 adults with Low and High Health Literacy on the four questions to assess health numeracy

No	Item question	Correct answer	
		Low health literacy (*N*, %)	High health literacy (*N*, %)
1	Interpret the dose as a fraction	98 (74)	149 (75)
2	Interpret the value of CD_4_ count	93 (70)	162 (81)
3	Interpret the date of the next clinical visit presented in an appointment slip	75 (56)	112 (56)
4	Interpret proper time to take a medication using written instructions	90 (68)	143 (72)

The median (IQR) of the domains distancing and blame of the HIV stigma scale, in the high health literacy group was higher than those in the low health literacy group, but those differences were not statistically significant. Table 3 also shows that PLHIV with low health literacy had a higher median in all domains of the BMQ than those with high literacy with three domains showing significant differences, i.e. PLHIV with low health literacy had more concerns (16.5 ± 5.0) were more worried about overuse (13.0 ± 4.0), and harmful effects (10.0 ± 5.0) than PLHIV with high health literacy (Table 3). There were no differences in perceived stigma between PLHIV with low and high health literacy (Table 3).

**TABLE 3:** Scale score medians and IQR for the subscales used to assess participant's Stigma and Beliefs about Medicines (BMQ) for PLHIV with low and high health literacy

Variable	Low health literacy Median ± IQR	High health literacy Median ± IQR	Mann–Whitney *U* test
Stigma subscale			
Distancing	8.0 ± 6.0	9.0 ± 7.0	.079
Blaming	7.5 ± 4.8	8.0 ± 4.0	.432
Discrimination	10.0 ± 6.0	10.0 ± 5.0	.267
BMQ subscale			
Necessity	19.0 ± 4.0	18.0 ± 4.0	.329
Concern	16.5 ± 5.0	16.0 ± 3.0	.003
Overuse	13.0 ± 4.0	11.0 ± 3.0	.000
Harm	10.0 ± 5.0	9.0 ± 3.0	.000

Abbreviations: IQR, inter quartile range.

In the multivariate logistic regression analysis type of dosing regimen, support in medicine‐taking, and two BMQ subscales, overuse and harm, were significantly associated with low health literacy. PLHIV with multi‐dose regimen were less likely to have low health literacy than those taking a fixed‐dose combination (OR = 0.51; 95%CI = 0.32–0.82). PLHIV who had social support in medicine‐taking were more likely to have low health literacy (OR = 1.78; 95%CI = 1.07–2.97). More awareness about medication overuse (OR = 1.17; 95%CI = 1.06–1.29) and medication harm (OR = 1.10; 95%CI = 1.01–1.20) were also associated with having low health literacy (Table 4).

**TABLE 4:** Results of univariate and multivariate analyses on the association of variables with Low Health Literacy

Independent variables		Univariate OR (95% CI)	*p*‐value	Multivariate AOR (95% CI)	*p*‐value
Gender	Male	Ref	.054		
	Female	0.64 (0.40–1.00)			
Ethnicity	Papuan	Ref	.192		
	Non‐Papuan	0.72 (0.45–1.17)			
Type of ART	Fixed‐dose Combination (FDC)	Ref	.009	Ref	.010
	Multi‐dose regimen	0.55 (0.98–2.55)		0.51 (0.32–0.82)	
Medicine‐taking support	No	Ref	.056	Ref	.026
	Yes	1.58 (0.35–0.86)		1.78 (1.07–2.97)	
HIV‐Stigma	Distancing	0.95 (0.89–1.01)	.079		
BMQ	Concern	1.11 (1.04–1.20)	.003		
	Overuse	1.21 (1.11–1.32)	.000	1.17 (1.06–1.29)	.001
	Harm	1.16 (1.08–1.26)	.000	1.10 (1.01–1.20)	.044

Abbreviations: AOR, adjusted odds ratio; BMQ, beliefs about medicines; CI, confidence interval; FDC, fixed‐dose combination; OR, odds ratio.

Adjusted model: Low Health Literacy = ethnicity + sex +ART regimen + social support + distancing +concern + overuse +harm.

## 4 DISCUSSION

Our cross‐sectional study of PLHIV who were on ART for at least 6 months in two hospitals in Jayapura showed that one of three PLHIV had low health literacy. The percentage of PLHIV with low health literacy is high, compared to other studies which use even higher cut‐off point in S‐TOFHLA assessment (Colbert, Sereika, & Erlen, 2013; Navarra, Nelson, & Larson, 2014). Our findings showed that health literacy was significantly associated with the absence of medicine‐taking support, being on a multi‐dose regimen of ART, and beliefs about overuse as well as harmful effects of medicines. Based on our knowledge, this was the first study that examined health literacy among PLHIV in a developing country in Asia (Rajah et al., 2019).

PLHIV with low health literacy were more likely to have medicine‐taking support. In line with a prior study, this may be positive in showing that PLHIV with low level of health literacy seek the support they need to cope with the daily tasks of medicine‐taking (Xu et al., 2017). In a wider context, it has been shown that having emotional support improves involvement in decision‐making, especially among people with lower education (Brabers, Jong, Groenewegen, & Dijk, 2016). Therefore, the presence of social support among PLHIV may not merely be there to remind them to take their medicine, but may be also directed towards making appropriate health decisions. Unfortunately, we did not assess the health literacy level of their social supports since there are prior studies showing that health literacy of social support influences the decision‐making process of PLHIV (Yuen, Knight, Ricciardelli, & Burney, 2018).

We find it difficult to explain one of our findings, which stated that PLHIV with low health literacy were more likely to be on a fixed‐dose combination of ART. In our setting, the fixed‐dose combinations are more expensive than the multi‐dose regimens. Therefore, the national guideline recommends that only PLHIV with co‐morbidities should receive fixed‐dose combinations to reduce the tablet‐burden (Kementerian Kesehatan RI, 2014). Since it was not possible to collect sufficient information on co‐morbidity, we could not investigate this association. In general, being on a fixed‐dose combination has been found to be positive for adherence (Galen, Nellen, & Nieuwkerk, 2014), due to the simplicity of the regimen (Libby et al., 2013) and better virological suppression (Nachega et al., 2014). We assume the simplification of ART by giving fixed‐dose combination might still leave our participants with a considerable burden of drugs for their co‐morbidities (Wawrzyniak & Ownby, 2013). It could be that health providers prescribed fixed‐dose combinations to people with low health literacy to improve adherence. Further investigation is needed to explore this association.

The last finding in this study showed that PLHIV with low health literacy had more worries about the overuse and harm of medication. Our results are in line with two previous studies in very different patient populations which also found that patients with low health literacy had more negative perceptions towards medications (Duggan et al., 2014; Kale et al., 2015). In general, negative beliefs about medicines are associated with low medication adherence (Horne et al., 2013).

Our study adds to the literature on health literacy among PLHIV and suggests that care should be tailored to the level of health literacy. Health professionals should be especially aware of negative perceptions about medications. Targeting some of those beliefs may improve medication use and self‐management behaviour. It may need more time and efforts to explain the use of a multi‐dose regimen as well as discuss concerns about medications with PLHIV with low health literacy. Simple media such as leaflets that contain printed words accompanied by pictures might be a reliable tool to increase awareness since 93% of Indonesian are literate (Wali, Hudani, Wali, Mercer, & Grindrod, 2016). Providing information stepwise and repeating information over time may be important additional aspects. Pharmacists or pharmacy assistants should take a role in this (Wali et al., 2016).

Our study has a number of limitations. We only included participants from two hospitals, so our results may not be generalisable to other areas in Indonesia. Furthermore, the cross‐sectional study limits causal inferences. Finally, we could only collect limited clinical data due to incomplete medical records, as is often the case in rural health centres in developing countries. Further work is needed to unravel the link between health literacy and clinical outcomes in PLHIV.

In conclusions, more than a third of PLHIV had a low level of health literacy. Social support and giving a fixed‐dose combination seems to be important for PLHIV with low health literacy. Health providers should tailor their care to address, concerns about medication overuse and harm, especially in PLHIV with low health literacy. A health literacy intervention for patients and their family or caregivers may be a promising strategy to improve self‐management. Using concrete instructions and having regular meetings to discuss ART with pharmacists may be one way forward.

## CONFLICT OF INTEREST

There are no conflicts of interest relating to any of the authors.

## AUTHOR CONTRIBUTIONS

Conceived and designed the study: EIS, KT. Analysed the data: EIS, DPA, SNS, AI, KT. Wrote the paper: EIS, DPA, SNS, AI, KT.

## References

[hsc13075-bib-0001] Asosiasi Penyelenggara Jasa Internet Indonesia (2017). Penetrasi & Prilaku Pengguna Internet Indonesia.

[hsc13075-bib-0002] Brabers, A. E. M. , de Jong, J. D. , Groenewegen, P. P. , & van Dijk, L. (2016). Social support plays a role in the attitude that people have towards taking an active role in medical decision‐making. BMC Health Services Research, 16(1), 1–11. 10.1186/s12913-016-1767-x 27655113 PMC5031318

[hsc13075-bib-0003] Colbert, A. M. , Sereika, S. M. , & Erlen, J. A. (2013). Functional health literacy, medication‐taking self‐efficacy and adherence to antiretroviral therapy. Journal of Advanced Nursing, 69(2), 295–304. 10.1111/j.1365-2648.2012.06007.x 22489684

[hsc13075-bib-0004] Davis, T. C. , Wolf, M. S. , Bass, P. F. , Thompson, J. A. , Tilson, H. H. , Neuberger, M. , & Parker, R. M. (2006). Literacy and misunderstanding prescription drug labels. Annals of Internal Medicine, 145(12), 887–895. 10.7326/0003-4819-145-12-200612190-00144 17135578

[hsc13075-bib-0005] Duggan, L. , McCarthy, S. , Curtis, L. M. , Wolf, M. S. , Noone, C. , Higgins, J. R. , … Sahm, L. J. (2014). Associations between health literacy and beliefs about medicines in an Irish obstetric population. Journal of Health Communication, 19(3), 106–114. 10.1080/10810730.2014.936570 25315587

[hsc13075-bib-0006] Epstein, J. , Santo, R. M. , & Guillemin, F. (2015). A review of guidelines for cross‐cultural adaptation of questionnaires could not bring out a consensus. Journal of Clinical Epidemiology, 68(4), 435 10.1016/j.jclinepi.2014.11.021 25698408

[hsc13075-bib-0007] Gazmarariana, J. A. , Williams, M. V. , Peel, J. , Baker, D. W. , Gazmararian, J. A. , Williams, M. V. , … Baker, D. W. (2003). Health literacy and knowledge of chronic disease. Patient Education and Counseling, 51(3), 267–275. 10.1016/S0738-3991(02)00239-2 14630383

[hsc13075-bib-0008] Horne, R. , Chapman, S. C. E. , Parham, R. , Freemantle, N. , Forbes, A. , & Cooper, V. (2013). Understanding patients’ adherence‐related Beliefs about Medicines prescribed for long‐term conditions: A meta‐analytic review of the Necessity‐Concerns Framework. PLoS One, 8(12), 10.1371/journal.pone.0080633 PMC3846635 24312488

[hsc13075-bib-0009] Horne, R. , & Weinman, J. (1999). Patients’beliefs about prescribed medicines and their role in adherence to treatment in chronic physical illness. Journal of Psychosomatic Research, 47(6), 555–568. 10.1016/S0022-3999(99)00057-4 10661603

[hsc13075-bib-0010] Indonesia S . (2013). Indonesia Demographic and Health Survey 2012. Retrieved from http://www.dhsprogram.com.

[hsc13075-bib-0011] Jordan, J. E. , Osborne, R. H. , & Buchbinder, R. (2011). Critical appraisal of health literacy indices revealed variable underlying constructs, narrow content and psychometric weaknesses. Journal of Clinical Epidemiology, 64(4), 366–379. 10.1016/j.jclinepi.2010.04.005 20638235

[hsc13075-bib-0012] Kale, M. S. , Federman, A. D. , Krauskopf, K. , Wolf, M. , O’Conor, R. , Martynenko, M. , … Wisnivesky, J. P. (2015). The association of health literacy with illness and medication beliefs among patients with chronic obstructive pulmonary disease. PLoS One, 10(4), 1–10. 10.1371/journal.pone.0123937 PMC4411058 25915420

[hsc13075-bib-0013] Kalichman, S. C. , Cherry, C. , Kalichman, M. O. , Amaral, C. , White, D. , Grebler, T. , … Schinazi, R. F. (2013). Randomized clinical trial of HIV treatment adherence counseling interventions for people living with HIV and limited health literacy. Journal of Acquired Immune Deficiency Syndromes, 63(1), 42–50. 10.1097/QAI.0b013e318286ce49.Randomized 23337369 PMC4279917

[hsc13075-bib-0014] Kementerian Kesehatan, R. I. (2014). Pedoman pengobatan antiretroviral. 10.1192/bjp.205.1.76a

[hsc13075-bib-0015] Kementerian Kesehatan Republik Indonesia ‐ Direktorat Jenderal Pengendalian Penyakit dan Penyehatan Lingkungan . (2011). Pedoman Nasional Tatalaksana Klinis Infeksi HIV dan Terapi Antiretroviral pada Orang Dewasa.

[hsc13075-bib-0016] Koirala, S. , Deuba, K. , Nampaisan, O. , Marrone, G. , & Ekstro, M. (2017). Facilitators and barriers for retention in HIV care between testing and treatment in Asia. PLoS ONE, 12(5), 1–20, 10.1371/journal.pone.0176914 PMC5411091 28459881

[hsc13075-bib-0017] Libby, A. M. , Fish, D. N. , Hosokawa, P. W. , Linnebur, S. A. , Metz, K. R. , Nair, K. V. , … Hirsch, J. D. (2013). Patient‐level medication regimen complexity across populations with chronic disease. Clinical Therapeutics, 35(4), 10.1016/j.clinthera.2013.02.019 23541707

[hsc13075-bib-0018] McCormack, L. , Squiers, L. , Boudewyns, V. , Berkman, N. , & Peinado, S. (2012). The health literacy skills framework. Journal of Health Communication, 17(sup3), 30–54. 10.1080/10810730.2012.713442 23030560

[hsc13075-bib-0019] Murray, C. , Ortblad, K. , Guinovart, C. , & Al, E. (2014). Global, regional, and national incidence and mortality for HIV, tuberculosis, and malaria during 1990–2013: A systematic analysis for the Global Burden of Disease Study 2013. Lancet, 20(384), 1005–1070. 10.1016/S0140-6736(14)60844-8.Global PMC4202387 25059949

[hsc13075-bib-0020] Nachega, J. B. , Parienti, J.‐J. , Uthman, O. A. , Gross, R. , Dowdy, D. W. , Sax, P. E. , … Giordano, T. P. (2014). Lower pill burden and once‐daily antiretroviral treatment regimens for HIV infection: A meta‐analysis of randomized controlled trials. Clinical Infectious Diseases, 58(9), 1297–1307. 10.1093/cid/ciu046 24457345 PMC3982838

[hsc13075-bib-0021] Navarra, A.‐M. , Neu, N. , Toussi, S. , Nelson, J. , & Larson, E. L. (2014). Health literacy and adherence to antiretroviral therapy among HIV‐infected youth. Journal of the Association of Nurses in AIDS Care, 25(3), 203–213. 10.1016/j.jana.2012.11.003 PMC3706514 23433916

[hsc13075-bib-0022] Palumbo, R. (2015). Discussing the effects of poor health literacy on patients facing HIV: A narrative literature review. International Journal of Health Policy and Management, 4(7), 417–430. 10.15171/ijhpm.2015.95. 10.15171/ijhpm.2015.95 26188806 PMC4493582

[hsc13075-bib-0023] Pane, M. , Sianturi, E. I. , Kong, Y. M. F. , Bautista, P. , & Taxis, K. (2018). Factors associated with regular counselling attendance of HIV outpatients of a national referral hospital in Jakarta, Indonesia: A cross sectional study. BMC Public Health, 18(1), 1–6. 10.1186/s12889-018-5924-5 PMC6102862 30126405

[hsc13075-bib-0024] Parker, R. M. , Baker, D. W. , Williams, M. V. , & Nurss, J. R. (1995). The test of functional health literacy in adults: A new instrument for measuring patients’ literacy skills. Journal of General Internal Medicine, 10(10), 537–541. Retrieved from http://www.ncbi.nlm.nih.gov/pubmed/8576769. 10.1007/BF02640361 8576769

[hsc13075-bib-0025] Pendse, R. , Gupta, S. , Yu, D. , & Sarkar, S. (2016). HIV/AIDS in the South‐East Asia region: Progress and challenges. Journal of Virus Eradication, 2(Suppl 4), 1–6. Retrieved from http://www.ncbi.nlm.nih.gov/pubmed/28303199%0Ahttp://www.pubmedcentral.nih.gov/articlerender.fcgi?artid=PMC5353351 10.1016/S2055-6640(20)31092-X PMC5353351 28303199

[hsc13075-bib-0026] Perazzo, J. , Haas, S. , Webel, A. , & Voss, J. (2017). Role of the internet in care initiation by people living with HIV. Research in Nursing and Health, 40(1), 43–50. 10.1002/nur.21769 27686871 PMC5484040

[hsc13075-bib-0028] Rajah, R. , Hassali, M. A. A. , & Murugiah, M. K. (2019). A systematic review of the prevalence of limited health literacy in Southeast Asian countries. Public Health, 167, 8–15. 10.1016/j.puhe.2018.09.028 30544041

[hsc13075-bib-0029] Raynor, D. K. T. (2012). Health literacy: Is it time to shift our focus from patient to provider? BMJ, 344(7852), 1–2. 10.1136/bmj.e2188

[hsc13075-bib-0030] Sharma, B. , & Jain, R. (2014). Right choice of a method for determination of cut‐off values: A statistical tool for a diagnostic test. Asian Journal of Medical Sciences, 5(3), 30–34.

[hsc13075-bib-0031] Sianturi, E. I. , Perwitasari, D. A. , Islam, A. , & Taxis, K. (2019). The association between ethnicity, stigma, beliefs about medicines and adherence in people living with HIV in a rural area in Indonesia. BMC Public Health, 1–8. 10.1186/s12889-019-6392-2 30634953 PMC6330480

[hsc13075-bib-0032] Sørensen, K. , Van den Broucke, S. , Fullam, J. , Doyle, G. , Pelikan, J. , Slonska, Z. , … Brand, H. (2012). Health literacy and public health: A systematic review and integration of definitions and models. BMC Public Health, 12(80), 80 10.1186/1471-2458-12-80 22276600 PMC3292515

[hsc13075-bib-0033] Sowell, R. L. , Lowenstein, A. , Moneyham, L. , Demi, A. , Mizuno, Y. , & Seals, B. F. (1997). Resources, stigma, and patterns of disclosure in rural women with HIV infection. Public Health Nursing, 14(5), 302–312. 10.1111/j.1525-1446.1997.tb00379.x 9342922

[hsc13075-bib-0034] Van Galen, K. A. , Nellen, J. F. , & Nieuwkerk, P. T. (2014). The effect on treatment adherence of administering drugs as fixed‐dose combinations versus as separate pills: Systematic review and meta‐analysis. AIDS Research and Treatment, 2014, 1–6. 10.1155/2014/967073 PMC4168145 25276422

[hsc13075-bib-0035] van Luenen, S. , Garnefski, N. , Spinhoven, P. , & Kraaij, V. (2018). Guided internet‐based intervention for people with HIV and depressive symptoms: A randomised controlled trial in the Netherlands. The Lancet HIV, 5(9), e488–e497. 10.1016/S2352-3018(18)30133-4 30135045

[hsc13075-bib-0036] Waite, K. R. , Paasche‐Orlow, M. , Rintamaki, L. S. , Davis, T. C. , & Wolf, M. S. (2008). Literacy, social stigma, and HIV medication adherence. Journal of General Internal Medicine, 23(9), 1367–1372. 10.1007/s11606-008-0662-5 18563494 PMC2518013

[hsc13075-bib-0037] Waldrop‐Valverde, D. , Jones, D. L. , Jayaweera, D. , Gonzalez, P. , Romero, J. , & Ownby, R. L. (2009). Gender differences in medication management capacity in HIV infection: The role of health literacy and numeracy. AIDS and Behavior, 13(1), 46–52. 10.1007/s10461-008-9425-x.Gender 18618237 PMC2606913

[hsc13075-bib-0038] Wali, H. , Hudani, Z. , Wali, S. , Mercer, K. , & Grindrod, K. (2016). A systematic review of interventions to improve medication information for low health literate populations. Research in Social and Administrative Pharmacy, 12(6), 830–864. 10.1016/j.sapharm.2015.12.001 26926671

[hsc13075-bib-0039] Ware, N. C. , Idoko, J. , Kaaya, S. , Biraro, I. A. , Wyatt, M. A. , Agbaji, O. , … Bangsberg, D. R. (2009). Explaining adherence success in sub‐Saharan Africa: An ethnographic study. PLoS Medicine, 6(1), 39–47. 10.1371/journal.pmed.1000011 PMC2631046 19175285

[hsc13075-bib-0040] Wawrzyniak, A. J. , & Ownby, R. L. (2013). Health literacy: Impact on the health of HIV‐infected individuals. Curr HIV/AIDS, 10(4), 295–304. 10.1007/s11904-013-0178-4.Health PMC4022478 24222474

[hsc13075-bib-0041] Wijayanti, F. , Tarmizi, S. N. , Tobing, V. , Nisa, T. , Akhtar, M. , Trihandini, I. , & Djuwita, R. (2016). From the millennium development goals to sustainable development goals. The response to the HIV epidemic in Indonesia: Challenges and opportunities. Journal of Virus Eradication, 2(Supplement 4), 27–31. 28275447 10.1016/S2055-6640(20)31096-7 PMC5337410

[hsc13075-bib-0042] Xu, J. F. , Ming, Z. Q. , Zhang, Y. Q. , Wang, P. C. , Jing, J. , & Cheng, F. (2017). Family support, discrimination, and quality of life among ART‐treated HIV‐infected patients: A two‐year study in China. Infectious Diseases of Poverty, 6(1), 1–10. 10.1186/s40249-017-0364-5 29157301 PMC5697335

[hsc13075-bib-0043] Yuen, E. Y. N. , Knight, T. , Ricciardelli, L. A. , & Burney, S. (2018). Health literacy of caregivers of adult care recipients: A systematic scoping review. Health and Social Care in the Community, 26(2), e191–e206. 10.1111/hsc.12368 27426731

[hsc13075-bib-0044] Zukoski, A. P. , Thorburn, S. , & Stroud, J. (2011). Seeking information about HIV/AIDS: A qualitative study of health literacy among people living with HIV/AIDS in a low prevalence context. AIDS Care, 23(11), 1505–1508. 10.1080/09540121.2011.582077 22022854

